# 9-(4-Meth­oxy­phen­yl)-9*H*-carbazole

**DOI:** 10.1107/S2414314623006740

**Published:** 2023-08-10

**Authors:** Prabhu Ganesan, Nithianantham Jeeva Jasmine, Robert Swinton Darious, Krishnan Soundararajan, Renganathan Rajalingam

**Affiliations:** a School of Chemistry, Bharathidasan University, Tiruchirappalli-620 024, Tamil Nadu, India; b Molecular Biophysics Unit, Indian Institute of Science, Bangalore, India; c PG & Research Department of Chemistry, Bishop Heber College (Autonomous), Tiruchirappalli-620 017, Tamil Nadu, India; d Department of Chemistry, Periyar Maniammai Institute of Science and Technology, Vallam-613403, Thanjavur, Tamil Nadu, India; University of Aberdeen, United Kingdom

**Keywords:** carbazole, dihedral angle, torsion angle, Hirshfeld surface analysis, crystal structure

## Abstract

In the title compound, C_19_H_15_NO, the dihedral angle between the benzene rings of the carbazole moiety is 1.73 (12)° and the meth­oxy-substituted phenyl ring deviates from the mean plane of the carbazole grouping (r.m.s. deviation = 0.020 Å) by 56.78 (8)°. In the crystal, weak C—H⋯π inter­actions link the mol­ecules. The two-dimensional fingerprint plots derived from the Hirshfeld surface indicate that H⋯H (51.2%) and C⋯H/H⋯C (39.9%) contacts dominate the packing.

## Abstract 2

In the title carbazole derivative, the dihedral angle between the carbazole ring system and the pendant phenyl ring is 56.78 (8)°.

## Graphical abstract

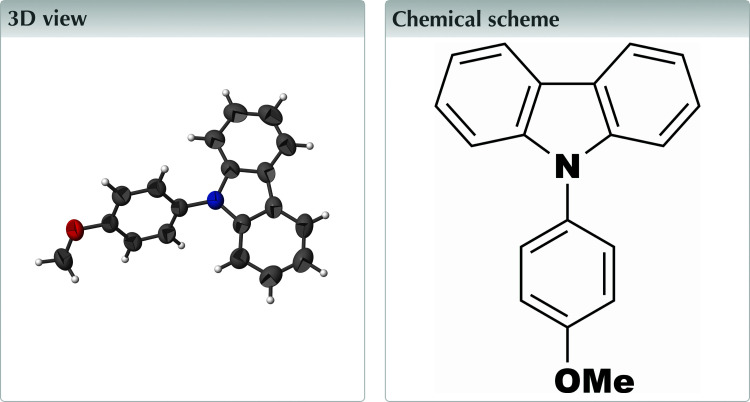

## Structure description

Carbazole and its derivatives have attracted attention in the development of electrical and electronic materials because of their conjugated π-electron systems (Taranekar *et al.*, 2007 ▸). The N-heterocyclic carbazole mol­ecule has also been employed as a promising candidate in the treatment of cancer (Patil *et al.*, 2022 ▸). As part of our studies in this area, we now describe the synthesis and structure of the title compound.

**Figure 1:**
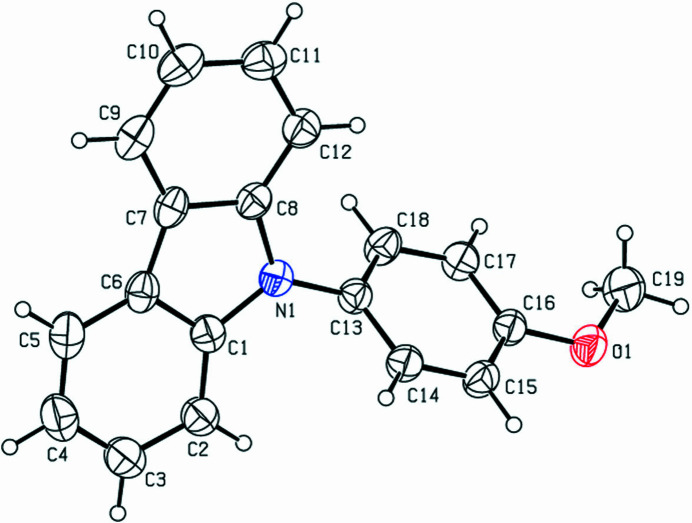
The mol­ecular structure of the title compound, showing displacement ellipsoids drawn at the 30% probability level.

**Figure 2:**
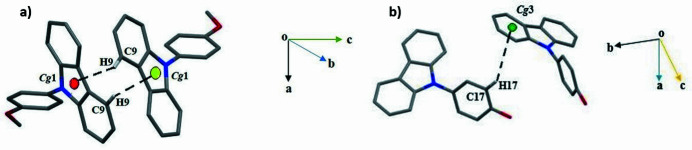
A view of the C—H⋯π inter­actions in the title compound. *Cg*1 is the centroid of the pyrrole ring of the carbazole mol­ecule (symmetry code: 1 − *x*, − 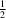 + *y*, 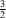 − *z*) and *Cg*3 is the centroid of the phenyl ring of the carbazole mol­ecule (symmetry code: *x*, 1 + *y*, *z*).

**Figure 3:**
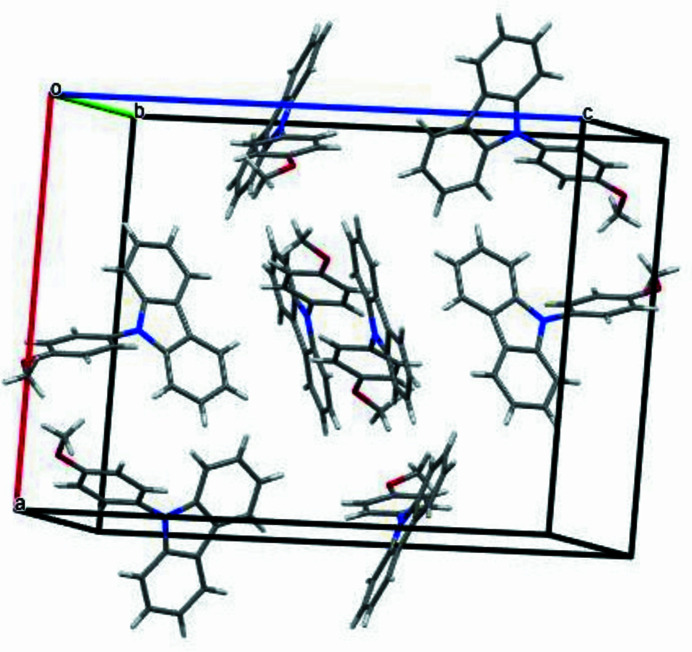
Crystal packing viewed along the *a*-axis direction.

The title compound crystallizes in the ortho­rhom­bic space group *Pbca* with one mol­ecule in the asymmetric unit (Fig. 1 ▸). As expected, the C1–C12/N1 carbazole-fused-ring moiety is almost planar (Moreno-Fuquen *et al.*, 2012 ▸) with a dihedral angle of 1.73 (12)° between the C1–C6 and C7–C12 rings. The dihedral angle between the C1–C12/N1 carbazole mean plane and the pendant C13–C18 ring is 56.78 (8)°. Atom C19 of the *para*-meth­oxy group deviates by 0.219 (3) Å from its attached ring. The crystal structure is consolidated by weak C—H⋯π inter­actions (Fig. 2 ▸) [C9—H9⋯*Cg*(N1/C1/C6–C8) = 2.71 Å; symmetry code: 1 − *x*, − 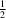 + *y*, 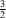 − *z*; C17—H17⋯*Cg*(C7–C12) = 2.92 Å; symmetry code: *x*, 1 + *y*, *z*]. The crystal packing viewed along the *a*-axis direction is shown in Fig. 3 ▸.

**Figure 4:**
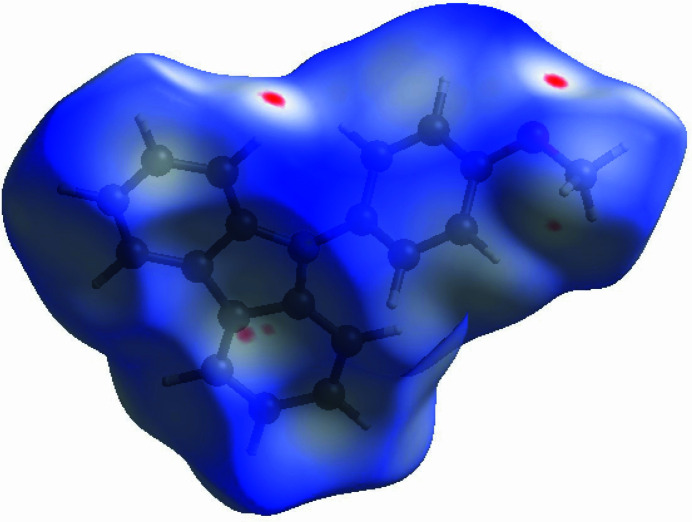
A view of the three-dimensional Hirshfeld surface of the title compound mapped over *d*_norm_ in the range −0.05 to 1.63 a.u.

**Figure 5:**
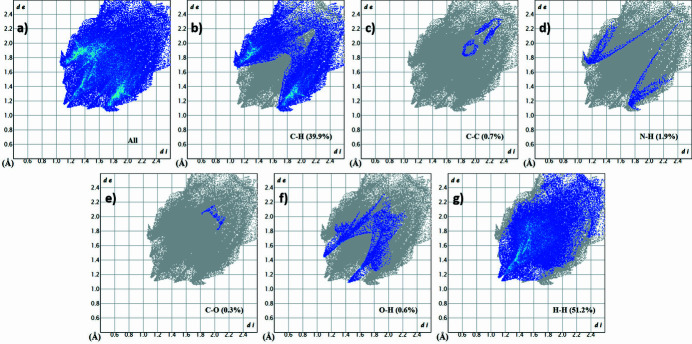
The two-dimensional fingerprint plots (*a*–*g*) showing all inter­molecular inter­actions and delineated into C⋯H/H⋯C, C⋯C, N⋯H/H⋯N, O⋯C/C⋯O, O—H⋯H⋯O and H⋯H contacts.

The Hirshfeld surface and its related two-dimensional fingerprint plots (Spackman & Jayatilaka, 2009 ▸) were generated with Crystal Explorer 17.5 (Turner *et al.*, 2017 ▸). The Hirshfeld surface mapped over *d*_norm_ within the range −0.05 to 1.63 a.u. shows a few red spots in the locales of *D*⋯*A* (*D* = donor, *A* = acceptor) inter­actions, consistent with the presence of weak C—H⋯π inter­actions (Fig. 4 ▸). The two-dimensional fingerprint plots (Fig. 5 ▸) show that H⋯H (51.2%) and C⋯H/H⋯C (39.9%) contacts dominate the packing with other contacts [C⋯C (0.7%), N⋯H/H⋯N (1.9%), O⋯C/C⋯O (0.3%), O⋯H/H⋯O (6.0%)] making minor contributions.

## Synthesis and crystallization

**Figure 6:**
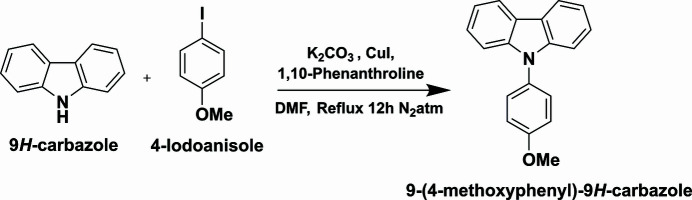
Reaction scheme.

A 100 ml round-bottom flask was charged with 4-iodo­anisole 1.78 g (7.6 mmol, 1 equiv), 9*H*-carbazole 1.143 g (6.8 mmol, 1.1 equiv), which were uniformly dispersed in 35 ml of di­methyl­formamide (DMF) and the mixture was dissolved homogeneously under an N_2_ atm. To the mixture, K_2_CO_3_ (5.25 g, 38 mmol, 5 equiv), CuI (0.144 g, 0.76 mmol, 0.1 equiv) and 1,10-phenanthroline (0.137 g, 0.76 mmol, 0.1 equiv) was added and the mixture was refluxed for 12 h under N_2_. The progress of the reaction was monitored by TLC. After the completion of the reaction, the reaction was quenched in ice–water and the solid product was dissolved in ethyl acetate and washed with brine solution. The obtained solvent was removed under reduced pressure and the obtained residue was further purified using column chromatography (100–200 mesh silica gel) to afford the title compound as shown in Fig. 6 ▸. Single crystals in the form of colourless needles were grown from di­chloro­methane solution at room temperature.

## Refinement

Crystal data, data collection and structure refinement details are summarized in Table 1 ▸.

## Supplementary Material

Crystal structure: contains datablock(s) I. DOI: 10.1107/S2414314623006740/hb4441sup1.cif

Structure factors: contains datablock(s) I. DOI: 10.1107/S2414314623006740/hb4441Isup2.hkl

Supporting information file. DOI: 10.1107/S2414314623006740/hb4441Isup3.cml

CCDC reference: 2280833

Additional supporting information:  crystallographic information; 3D view; checkCIF report

## Figures and Tables

**Table 1:** Experimental details

Crystal data	
Chemical formula	C_19_H_15_NO
*M*_r_	273.32
Crystal system, space group	Orthorhombic, *P* *b* *c* *a*
Temperature (K)	300
*a*, *b*, *c* (Å)	16.2645 (16), 7.8297 (7), 22.819 (2)
*V* (Å^3^)	2905.9 (5)
*Z*	8
Radiation type	Mo *K*α
μ (mm^−1^)	0.08
Crystal size (mm)	0.30 × 0.09 × 0.07

Data collection	
Diffractometer	Bruker D8 CCD
Absorption correction	Multi-scan (*SADABS*; Krause *et al.*, 2015 ▸)
*T*_min_, *T*_max_	0.665, 0.745
No. of measured, independent and observed [*I* > 2σ(*I*)] reflections	23109, 2762, 1564
*R*_int_	0.062
(sin θ/λ)_max_ (Å^−1^)	0.610

Refinement	
*R*[*F* ^2^ > 2σ(*F* ^2^)], *wR*(*F* ^2^), *S*	0.046, 0.136, 1.08
No. of reflections	2762
No. of parameters	192
H-atom treatment	H-atom parameters constrained
Δρ_max_, Δρ_min_ (e Å^−3^)	0.16, −0.16

Computer programs: *APEX4* and *SAINT* (Bruker, 2016 ▸), *SHELXT* (Sheldrick, 2015*a* ▸), *SHELXL2019/1* (Sheldrick, 2015*b* ▸) and *SHELXTL* (Sheldrick, 2008 ▸).

## full crystallographic data

### Crystal data

**Table:**

C_19_H_15_NO	*D*_x_ = 1.249 Mg m^−^^3^
*M**_r_* = 273.32	Mo *K*α radiation, λ = 0.71073 Å
Orthorhombic, *P**b**c**a*	Cell parameters from 4004 reflections
*a* = 16.2645 (16) Å	θ = 2.5–21.3°
*b* = 7.8297 (7) Å	µ = 0.08 mm^−^^1^
*c* = 22.819 (2) Å	*T* = 300 K
*V* = 2905.9 (5) Å^3^	Needle, colourless
*Z* = 8	0.30 × 0.09 × 0.07 mm
*F*(000) = 1152	

### Data collection

**Table:**

Bruker D8 CCD diffractometer	1564 reflections with *I* > 2σ(*I*)
Radiation source: i-mu-s microfocus source	*R*_int_ = 0.062
φ and ω scans	θ_max_ = 25.7°, θ_min_ = 2.2°
Absorption correction: multi-scan (SADABS; Krause *et al.*, 2015)	*h* = −17→19
*T*_min_ = 0.665, *T*_max_ = 0.745	*k* = −9→8
23109 measured reflections	*l* = −27→27
2762 independent reflections	

### Refinement

**Table:**

Refinement on *F*^2^	Hydrogen site location: inferred from neighbouring sites
Least-squares matrix: full	H-atom parameters constrained
*R*[*F*^2^ > 2σ(*F*^2^)] = 0.046	*w* = 1/[σ^2^(*F*_o_^2^) + (0.0466*P*)^2^ + 0.7157*P*] where *P* = (*F*_o_^2^ + 2*F*_c_^2^)/3
*wR*(*F*^2^) = 0.136	(Δ/σ)_max_ < 0.001
*S* = 1.08	Δρ_max_ = 0.16 e Å^−^^3^
2762 reflections	Δρ_min_ = −0.16 e Å^−^^3^
192 parameters	Extinction correction: *SHELXL2019/1* (Sheldrick, 2015b), Fc^*^=kFc[1+0.001xFc^2^λ^3^/sin(2θ)]^-1/4^
0 restraints	Extinction coefficient: 0.0059 (8)
Primary atom site location: dual	

### Special details

**Table:**

Geometry. All esds (except the esd in the dihedral angle between two l.s. planes) are estimated using the full covariance matrix. The cell esds are taken into account individually in the estimation of esds in distances, angles and torsion angles; correlations between esds in cell parameters are only used when they are defined by crystal symmetry. An approximate (isotropic) treatment of cell esds is used for estimating esds involving l.s. planes.

### Fractional atomic coordinates and isotropic or equivalent isotropic displacement parameters (Å^2^)

**Table:**

	*x*	*y*	*z*	*U*_iso_*/*U*_eq_	
O1	0.65084 (10)	0.6420 (2)	0.51323 (7)	0.0716 (5)	
N1	0.53757 (11)	0.0699 (2)	0.63305 (7)	0.0556 (5)	
C1	0.46058 (13)	−0.0072 (3)	0.62775 (9)	0.0549 (6)	
C2	0.39388 (14)	0.0412 (3)	0.59364 (10)	0.0635 (6)	
H2	0.395977	0.137531	0.569851	0.076*	
C3	0.32440 (16)	−0.0593 (4)	0.59644 (11)	0.0751 (7)	
H3	0.278853	−0.030830	0.573819	0.090*	
C4	0.32109 (18)	−0.2021 (4)	0.63231 (12)	0.0824 (8)	
H4	0.273344	−0.267428	0.633199	0.099*	
C5	0.38662 (17)	−0.2493 (3)	0.66653 (11)	0.0738 (7)	
H5	0.383598	−0.345344	0.690406	0.089*	
C6	0.45791 (14)	−0.1502 (3)	0.66481 (9)	0.0576 (6)	
C7	0.53597 (14)	−0.1588 (3)	0.69436 (9)	0.0570 (6)	
C8	0.58357 (14)	−0.0225 (3)	0.67375 (9)	0.0546 (6)	
C9	0.56892 (18)	−0.2696 (3)	0.73589 (10)	0.0705 (7)	
H9	0.537681	−0.359282	0.750651	0.085*	
C10	0.64787 (19)	−0.2448 (4)	0.75469 (11)	0.0795 (8)	
H10	0.670210	−0.317769	0.782615	0.095*	
C11	0.69510 (16)	−0.1116 (3)	0.73253 (11)	0.0746 (7)	
H11	0.748920	−0.098279	0.745508	0.089*	
C12	0.66401 (14)	0.0014 (3)	0.69172 (10)	0.0638 (6)	
H12	0.695869	0.090072	0.676892	0.077*	
C13	0.56442 (12)	0.2203 (3)	0.60315 (9)	0.0516 (6)	
C14	0.56432 (13)	0.2272 (3)	0.54252 (9)	0.0586 (6)	
H14	0.545321	0.134254	0.521051	0.070*	
C15	0.59196 (14)	0.3697 (3)	0.51393 (9)	0.0611 (6)	
H15	0.591165	0.373799	0.473205	0.073*	
C16	0.62103 (12)	0.5076 (3)	0.54551 (9)	0.0537 (6)	
C17	0.62057 (13)	0.5029 (3)	0.60578 (10)	0.0592 (6)	
H17	0.639820	0.595723	0.627203	0.071*	
C18	0.59139 (14)	0.3598 (3)	0.63427 (9)	0.0601 (6)	
H18	0.589963	0.357611	0.675011	0.072*	
C19	0.68915 (18)	0.7775 (3)	0.54377 (13)	0.0909 (9)	
H19A	0.711215	0.857935	0.516175	0.136*	
H19B	0.732795	0.733090	0.567635	0.136*	
H19C	0.649438	0.833600	0.568244	0.136*	

### Atomic displacement parameters (Å^2^)

**Table:**

	*U*^11^	*U*^22^	*U*^33^	*U*^12^	*U*^13^	*U*^23^
O1	0.0844 (11)	0.0660 (11)	0.0644 (10)	−0.0169 (9)	0.0010 (8)	0.0117 (9)
N1	0.0580 (12)	0.0545 (11)	0.0542 (11)	−0.0044 (10)	0.0001 (9)	0.0066 (9)
C1	0.0562 (14)	0.0549 (14)	0.0536 (13)	−0.0036 (12)	0.0058 (11)	−0.0073 (11)
C2	0.0610 (15)	0.0695 (16)	0.0602 (14)	−0.0034 (13)	0.0033 (12)	−0.0064 (12)
C3	0.0639 (17)	0.093 (2)	0.0688 (16)	−0.0055 (15)	0.0041 (13)	−0.0164 (16)
C4	0.0749 (19)	0.096 (2)	0.0758 (18)	−0.0262 (16)	0.0184 (16)	−0.0219 (17)
C5	0.0855 (19)	0.0682 (16)	0.0678 (17)	−0.0190 (15)	0.0196 (15)	−0.0063 (14)
C6	0.0686 (16)	0.0521 (14)	0.0521 (13)	−0.0033 (12)	0.0149 (12)	−0.0058 (11)
C7	0.0701 (16)	0.0509 (13)	0.0500 (12)	0.0042 (12)	0.0148 (12)	0.0004 (11)
C8	0.0606 (14)	0.0560 (14)	0.0473 (12)	0.0053 (12)	0.0051 (11)	0.0015 (11)
C9	0.095 (2)	0.0605 (16)	0.0564 (15)	0.0038 (14)	0.0129 (14)	0.0084 (12)
C10	0.100 (2)	0.0733 (18)	0.0646 (16)	0.0181 (17)	−0.0011 (15)	0.0085 (14)
C11	0.0751 (17)	0.0827 (19)	0.0658 (16)	0.0164 (15)	−0.0052 (13)	−0.0009 (15)
C12	0.0655 (16)	0.0662 (15)	0.0596 (14)	0.0036 (13)	0.0039 (12)	0.0032 (13)
C13	0.0537 (13)	0.0495 (13)	0.0517 (13)	−0.0010 (10)	0.0008 (10)	0.0035 (11)
C14	0.0675 (15)	0.0564 (14)	0.0519 (13)	−0.0082 (12)	0.0000 (11)	−0.0044 (11)
C15	0.0713 (16)	0.0668 (16)	0.0452 (13)	−0.0062 (13)	0.0025 (11)	−0.0006 (12)
C16	0.0533 (13)	0.0542 (14)	0.0538 (14)	−0.0003 (11)	0.0036 (10)	0.0070 (12)
C17	0.0712 (15)	0.0509 (14)	0.0557 (14)	−0.0066 (12)	0.0018 (11)	−0.0055 (12)
C18	0.0753 (16)	0.0578 (14)	0.0471 (12)	−0.0026 (13)	0.0034 (11)	−0.0008 (12)
C19	0.113 (2)	0.0664 (18)	0.093 (2)	−0.0338 (16)	−0.0148 (17)	0.0135 (16)

### Geometric parameters (Å, º)

**Table:**

O1—C16	1.373 (2)	C9—H9	0.9300
O1—C19	1.414 (3)	C10—C11	1.390 (3)
N1—C8	1.395 (3)	C10—H10	0.9300
N1—C1	1.396 (3)	C11—C12	1.380 (3)
N1—C13	1.429 (3)	C11—H11	0.9300
C1—C2	1.388 (3)	C12—H12	0.9300
C1—C6	1.404 (3)	C13—C18	1.374 (3)
C2—C3	1.379 (3)	C13—C14	1.385 (3)
C2—H2	0.9300	C14—C15	1.369 (3)
C3—C4	1.387 (4)	C14—H14	0.9300
C3—H3	0.9300	C15—C16	1.381 (3)
C4—C5	1.372 (4)	C15—H15	0.9300
C4—H4	0.9300	C16—C17	1.376 (3)
C5—C6	1.395 (3)	C17—C18	1.380 (3)
C5—H5	0.9300	C17—H17	0.9300
C6—C7	1.439 (3)	C18—H18	0.9300
C7—C9	1.392 (3)	C19—H19A	0.9600
C7—C8	1.400 (3)	C19—H19B	0.9600
C8—C12	1.384 (3)	C19—H19C	0.9600
C9—C10	1.368 (3)		
			
C16—O1—C19	117.80 (18)	C9—C10—H10	119.6
C8—N1—C1	108.32 (17)	C11—C10—H10	119.6
C8—N1—C13	125.53 (18)	C12—C11—C10	121.6 (2)
C1—N1—C13	126.13 (18)	C12—C11—H11	119.2
C2—C1—N1	129.2 (2)	C10—C11—H11	119.2
C2—C1—C6	122.1 (2)	C11—C12—C8	117.4 (2)
N1—C1—C6	108.73 (19)	C11—C12—H12	121.3
C3—C2—C1	117.3 (2)	C8—C12—H12	121.3
C3—C2—H2	121.3	C18—C13—C14	119.1 (2)
C1—C2—H2	121.3	C18—C13—N1	120.36 (18)
C2—C3—C4	121.3 (3)	C14—C13—N1	120.57 (19)
C2—C3—H3	119.4	C15—C14—C13	120.5 (2)
C4—C3—H3	119.4	C15—C14—H14	119.8
C5—C4—C3	121.5 (2)	C13—C14—H14	119.8
C5—C4—H4	119.2	C14—C15—C16	120.1 (2)
C3—C4—H4	119.2	C14—C15—H15	120.0
C4—C5—C6	118.7 (2)	C16—C15—H15	120.0
C4—C5—H5	120.7	O1—C16—C17	123.9 (2)
C6—C5—H5	120.7	O1—C16—C15	116.10 (19)
C5—C6—C1	119.1 (2)	C17—C16—C15	119.9 (2)
C5—C6—C7	133.9 (2)	C16—C17—C18	119.6 (2)
C1—C6—C7	106.99 (19)	C16—C17—H17	120.2
C9—C7—C8	119.4 (2)	C18—C17—H17	120.2
C9—C7—C6	133.5 (2)	C13—C18—C17	120.8 (2)
C8—C7—C6	107.15 (19)	C13—C18—H18	119.6
C12—C8—N1	129.4 (2)	C17—C18—H18	119.6
C12—C8—C7	121.8 (2)	O1—C19—H19A	109.5
N1—C8—C7	108.81 (19)	O1—C19—H19B	109.5
C10—C9—C7	119.1 (2)	H19A—C19—H19B	109.5
C10—C9—H9	120.4	O1—C19—H19C	109.5
C7—C9—H9	120.4	H19A—C19—H19C	109.5
C9—C10—C11	120.7 (2)	H19B—C19—H19C	109.5
			
C8—N1—C1—C2	178.5 (2)	C9—C7—C8—N1	−179.75 (18)
C13—N1—C1—C2	−0.1 (3)	C6—C7—C8—N1	0.4 (2)
C8—N1—C1—C6	−0.6 (2)	C8—C7—C9—C10	−1.4 (3)
C13—N1—C1—C6	−179.14 (18)	C6—C7—C9—C10	178.4 (2)
N1—C1—C2—C3	179.7 (2)	C7—C9—C10—C11	−0.4 (4)
C6—C1—C2—C3	−1.3 (3)	C9—C10—C11—C12	1.0 (4)
C1—C2—C3—C4	0.6 (3)	C10—C11—C12—C8	0.2 (3)
C2—C3—C4—C5	0.1 (4)	N1—C8—C12—C11	−179.1 (2)
C3—C4—C5—C6	0.0 (4)	C7—C8—C12—C11	−2.1 (3)
C4—C5—C6—C1	−0.7 (3)	C8—N1—C13—C18	−55.6 (3)
C4—C5—C6—C7	178.9 (2)	C1—N1—C13—C18	122.8 (2)
C2—C1—C6—C5	1.4 (3)	C8—N1—C13—C14	123.9 (2)
N1—C1—C6—C5	−179.44 (19)	C1—N1—C13—C14	−57.8 (3)
C2—C1—C6—C7	−178.32 (19)	C18—C13—C14—C15	1.1 (3)
N1—C1—C6—C7	0.8 (2)	N1—C13—C14—C15	−178.38 (19)
C5—C6—C7—C9	−0.3 (4)	C13—C14—C15—C16	0.7 (3)
C1—C6—C7—C9	179.5 (2)	C19—O1—C16—C17	5.2 (3)
C5—C6—C7—C8	179.6 (2)	C19—O1—C16—C15	−173.4 (2)
C1—C6—C7—C8	−0.7 (2)	C14—C15—C16—O1	177.25 (19)
C1—N1—C8—C12	177.4 (2)	C14—C15—C16—C17	−1.4 (3)
C13—N1—C8—C12	−4.1 (3)	O1—C16—C17—C18	−178.16 (19)
C1—N1—C8—C7	0.1 (2)	C15—C16—C17—C18	0.4 (3)
C13—N1—C8—C7	178.68 (18)	C14—C13—C18—C17	−2.1 (3)
C9—C7—C8—C12	2.7 (3)	N1—C13—C18—C17	177.35 (19)
C6—C7—C8—C12	−177.11 (19)	C16—C17—C18—C13	1.4 (3)
